# General Ward Nurses’ Self-Efficacy, Ethical Behavior, and Practice of Discharge Planning for End-Stage Cancer Patients: Path Analysis

**DOI:** 10.3390/healthcare10071161

**Published:** 2022-06-22

**Authors:** Michiko Aoyanagi, Yukari Shindo, Keita Takahashi

**Affiliations:** 1 Faculty of Health Sciences, Hokkaido University, Sapporo 060-0812, Japan; 2 Faculty of Health Sciences, Japan Health Care University, Sapporo 062-0053, Japan; y_shindo@jhu.ac.jp; 3 Institute of Health Science Innovation for Medical Care, Hokkaido University Hospital, Sapporo 060-8648, Japan; ktakahashi@pop.med.hokudai.ac.jp

**Keywords:** general ward nurses, end-stage cancer patients, self-efficacy, discharge planning, ethical behavior

## Abstract

General ward nurses play a key role in discharge planning for end-stage cancer patients. It is necessary to assess the factors regarding their practice to promote discharge planning in accordance with end-stage cancer patients’ wishes. This study aimed to investigate the relationships between general ward nurses’ practice of discharge planning for end-stage cancer patients, self-efficacy, ethical behavior, attitude, knowledge and experience, perceived skills, and perceived barriers. A total of 288 general ward nurses from nine hospitals in a city in Japan completed the questionnaire. Path analysis was conducted to test the hypotheses. The results showed that nurses’ self-efficacy, ethical behavior (do-no-harm, do-good), knowledge (experience of attending home care seminars), and perceived skills (assertiveness) were positively and directly related to the practice of discharge planning. Nursing experience and perceived skills (assertiveness) were positively associated with discharge planning practice, while perceived barriers (death discussion) and attitude (degree of leaving it to discharge planning nurses (DPNs)) were negatively associated, with self-efficacy acting as a mediator. Thus, our findings show that it is important to enhance self-efficacy and nursing ethical behavior to improve the practice of discharge planning. Accordingly, education regarding home care, assertive communication skills, death discussion, and ethics is needed for general ward nurses.

## 1. Introduction

The number of worldwide deaths resulting from cancer has been increasing annually. The International Agency for Research on Cancer [1] reported that 10 million people died from the disease in 2020. Nurses play a role in supporting cancer patients and their families in making decisions without regret until the patient’s passing. One of their important decisions is regarding where patients receive palliative care and where they die. Many end-stage cancer patients prefer to be cared for and die at home [2]. Some studies have shown that home-based death improves patients’ quality of life [3,4] and the quality of dying and death [5]. Cancer has a unique disease trajectory that differs from that of other diseases in that patients’ performance and self-care ability decline rapidly during their last few months [6]. Therefore, nurses are required to perform discharge planning before patients’ physical conditions decline rapidly. In this setting, general ward nurses should play a key role in such planning because it was shown that about 70% of cancer patients died in hospital general wards, other than palliative care wards, in Japan [7].

The need for early discharge planning has been emphasized in Japan, as in many other countries. Ward nurses should collaborate with discharge planning nurses (DPNs), who specialize in discharge planning in hospitals and coordinate homecare services [8]. DPNs begin discharge planning upon request from other health professionals, particularly general ward nurses [9]. Therefore, it is important that general ward nurses, who are in constant contact with end-stage cancer patients and their families, confirm their patients’ preferences for a place to receive end-of-life care and a place to die; understand their wishes regarding how to spend their remaining time; provide information regarding home care; and facilitate interprofessional collaboration to render their transition feasible. However, previous studies reported that general ward nurses’ discharge planning was insufficient [10,11]. It is necessary to improve nurses’ discharge planning to ensure a satisfying life for cancer patients in their end-stage. In order to achieve that, clarifying factors related to general nurses’ discharge planning might be helpful.

Attitude, knowledge, and skills are essential factors in nursing practice and a higher level of these help improve nursing practice. It was found that nurses had a perception that discharge planning was significant [12]. However, nurses also had a passive attitude as they thought discharge planning should be done by discharge liaison nurses [12]. Regarding knowledge, Yoshioka [13] reported that experience in supporting transitional care, longer years of nursing experience, and education on homecare nursing were related to higher support from nurses during the transition of end-stage cancer patients to homecare. Regarding skills, it was found that communication skills were important and had an impact on discharge planning practice [13]. General ward nurses’ discharge planning practice consists of understanding the intentions and wishes of patients and their families, providing information to them, supporting decision making, and cooperating with other healthcare providers [14,15]. To fulfill these roles, communication skills are indispensable. Especially in end-stage cancer patients’ discharge planning, it is vital for nurses, through communication, to draw out the wishes of patients and their families and support the process of deciding where the patient is to be cared for or die. Communication skills are important not only between patients and healthcare providers, including nurses, but also among healthcare providers when it comes to the discharge planning process [16].

Besides attitude, knowledge, and skills, previous studies show some barriers for discharge planning, such as communication barriers to discussing death with patients [17], a lack of time to prepare for discharge [17,18,19], and insufficient interprofessional collaboration [18]. Considering these barriers, nurses must promote the discharge planning of end-stage cancer patients, which requires clarification of the factors enabling nurses to promote it appropriately. Thus, we focused on nurses’ self-efficacy in facilitating discharge planning and preserving ethics to overcome barriers.

Bandura’s self-efficacy theory is well-known for promoting individuals’ behavior [20]. According to Bandura’s theory, self-efficacy refers to the belief in one’s own capabilities and mastery to perform. It influences a person’s choices and reactions to obstacles, and someone who has higher self-efficacy is more likely to behave better than someone with lower self-efficacy [21]. Based on this theory, nurses who have higher self-efficacy of discharge planning for end-stage cancer patients might perform better. Previous studies have shown that self-efficacy is a predictor [22,23,24,25] and mediator of nursing practice [23,25]. Nurses’ self-efficacy of practice is reportedly influenced by knowledge, nursing experience [26], perceived skills, perceived barriers, and teamwork beliefs [25]. Self-efficacy is also known to be correlated with attitudes [27].

Regarding nurses’ self-efficacy of discharge planning, Chaboyer et al. [28] evaluated the impact of ICU discharge liaison nurses’ intervention. They reported that there was no significant change of the nurses’ self-efficacy after the intervention. Hsu et al. [29] evaluated the effectiveness of discharge planning communication training for nurses using self-efficacy and reported that it was higher with simulation education than with conventional lectures. Thus, in previous studies, self-efficacy has been used as an indicator to evaluate the effectiveness of interventions and education regarding discharge planning. However, the actual situation and factors related to nurses’ self-efficacy of discharge planning for end-stage cancer patients and their impact on effective planning practice have not been sufficiently examined.

Beauchamp and Childress [30] suggested four principles of biomedical ethics: respect for autonomy, nonmaleficence, beneficence, and justice. Discharge planning practice for patients with end-stage cancer involves ethical issues because nurses should respect the patients’ autonomy in choosing their place of care while considering beneficence and nonmaleficence [31]. We can consider the discharge planning practice, including respect for patients’ autonomy and nurses’ behavior based on the beneficence and nonmaleficence principles, to be applicable to end-stage cancer patients. It is assumed that nurses with greater ethics provide better discharge planning. However, this relationship has not been assessed in previous studies.

To our knowledge, no previous studies have assessed the effects of self-efficacy and ethical principles, such as beneficence and nonmaleficence, on the practice of general ward nurses’ discharge planning for end-stage cancer patients. To fulfill end-stage cancer patients’ wishes to be cared for and die where they want, it is important to understand the variables related to the nurses’ discharge planning practice.

### Aims

**Figure 1:**
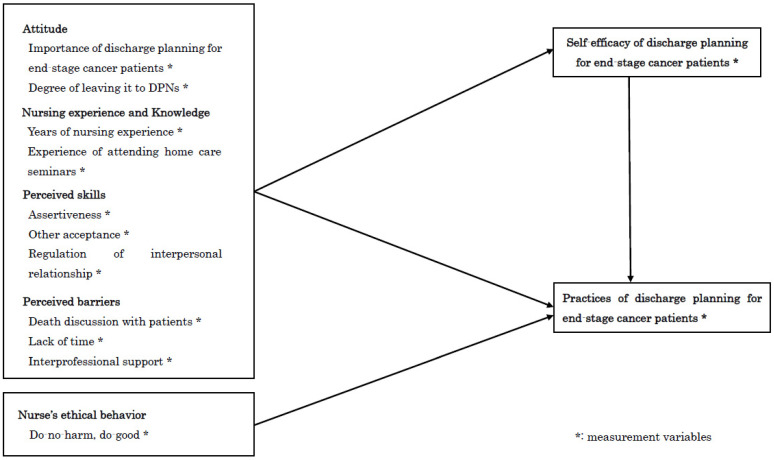
The hypothesized model.

A hypothesized model was constructed based on Bandura’s self-efficacy theory, factors related to discharge planning practice, and the findings of previous studies on self-efficacy (Figure 1).

This study aimed to examine a proposed model of the interrelationships among six factors (attitude, knowledge and nursing experience, perceived skills, perceived barriers, nurses’ ethical behavior, and self-efficacy) and the practice of discharge planning for end-stage cancer patients, using a path analysis. The six factors consisted of twelve values as follows: importance of discharge planning for end-stage cancer patients and degree of leaving it to DPNs (attitude); years of nursing experience and experience of attending homecare seminars (nursing experience and knowledge); assertiveness, other acceptance, and regulation of interpersonal relationships (perceived skills); death discussion with patients, lack of time, and interpersonal support (perceived barriers); do-no-harm, do-good (nurses’ ethical behavior); and self-efficacy regarding discharge planning for patients with end-stage cancer. Our hypothesized model was that: (1) attitude, nursing experience and knowledge, and perceived skills and perceived barriers would directly influence nurses’ self-efficacy of discharge planning for end-stage cancer patients; (2) self-efficacy would be the central factor, directly influencing practices of discharge planning for end-stage cancer patients and mediating the relationships between distal factors and discharge planning; (3) attitude, nursing experience and knowledge, and perceived skills and barriers would have a direct and mediatory influence on practices of discharge planning for end-stage cancer patients; and (4) nurses’ ethical behavior would directly influence their practices of discharge planning for end-stage cancer patients.

## 2. Materials and Methods

### 2.1. Setting and Study Design

A cross-sectional survey was conducted. We performed an exploratory path analysis based on the hypothesized model. We extended participation invitations to all 13 hospitals in Sapporo designated as facilities to promote home-based cancer and palliative care in the Hokkaido prefecture, Japan. Nine hospitals agreed to participate in this study. The individual participants were nurses working in the general wards of these nine hospitals, in which cancer patients were hospitalized. A self-administered questionnaire was distributed to the directors of nursing at the hospitals via mail, with their permission. In a structural equation model analysis, the desired sample size is over 200 [32,33]. We estimated that the response rate would be 60%, and the total number of self-administered questionnaires distributed was 475. The directors of nursing were asked to select the wards in which end-stage cancer patients were hospitalized and to distribute the questionnaires and cover letters to all the nurses at these wards. In the cover letter, nurses were asked to place the questionnaire into an envelope anonymously, seal it, and return it to their directors of nursing within two weeks using dedicated boxes. The nurses’ participation was voluntary. This survey was conducted between September and October 2018. The study was approved by the ethical review committee at the university with which the authors were affiliated.

### 2.2. Measures

The questionnaire included eight sections: demographic variables; nurses’ attitudes toward discharge planning for patients with end-stage cancer; nursing experience and knowledge; perceived skills; perceived barriers; nurses’ ethical behavior; self-efficacy; and discharge planning practice.

#### 2.2.1. Demographic Variables

Demographic variables, such as age, gender, educational background, ward setting, and whether the nurses had experience with homecare or DPNs, were collected.

#### 2.2.2. Attitude toward Discharge Planning for End-Stage Cancer Patients

We measured the nurses’ level of importance regarding discharge planning for patients with end-stage cancer and the level to which they would like to leave this discharge planning to DPNs. Participants were asked to evaluate the level of importance and the level of wanting to leave the task to DPNs using a visual analog scale (VAS). The VAS was a straight 100 mm line, which was anchored at each end (0 mm = “not important” to 100 mm = “extremely important”; 0 mm = “I do not want to leave discharge planning to DPNs at all” to 100 mm = “I want to leave discharge planning to DPNs”). The VAS is one of the common psychosocial measurement methods and has previously been used to measure attitude [34].

#### 2.2.3. Nursing Experience and Knowledge

Participants were asked about their years of nursing experience and whether they had experience with attending home care seminars.

#### 2.2.4. Perceived Skills

Skills were assessed as communication skills measured using the established ENDCOREs scale developed by Fujimoto, which has shown validity and reliability (goodness of fit index (GFI) = 0.98, adjusted goodness of fit index (AGFI) = 0.94, comparative fit index (CFI) = 0.97, root mean square error of approximation (RMSEA) = 0.08, and Cronbach’s αs were as follows: 0.80 for assertiveness, 0.83 for other acceptance, and 0.78 for regulation of interpersonal relationships) [35,36]. The scale includes six factors pertaining to communication skills, divided into basic and interpersonal levels. The current study used three factors at an interpersonal level: assertiveness, other acceptance, and regulation of interpersonal relationships. Each factor includes four items, such as “I explain my opinion in a logical way” (assertiveness), “I accept the opinions of others as much as possible” (other acceptance), and “I deal appropriately with discord due to disagreements” (regulation of interpersonal relationships). Responses are provided using a scale ranging from 1 (poor) to 7 (excellent). Higher scores reflect stronger communication skills. Cronbach’s αs in the current study were as follows: assertiveness: 0.89, other acceptance: 0.94, and regulation of interpersonal relationships: 0.87.

#### 2.2.5. Perceived Barriers

Barriers were assessed using three questions. Participants were asked to evaluate their practice levels of discussing death with patients, collaborating with an interprofessional team regarding patients’ care, and lack of time to prepare for discharge planning with the following possible responses: 1 = none, 2 = a little, 3 = quite a lot, 4 = a lot.

#### 2.2.6. Nurses’ Ethical Behavior

Nursing ethics were assessed using the Ethical Behavior Scale for Nurses developed by Ode [37]. The scale consists of three subscales: respect for the patient’s autonomy; justice for all patients; and do-no-harm, do-good. These scales were developed based on four principles of biomedical ethics, measuring the nurses’ self-perception of their ethical behavior. In the current study, only the do-no-harm, do-good scale was used, which was developed based on the nonmaleficence and beneficence principle. This scale consists of nine items. Examples of items include “I should always think of the best way to care for my patients” and “I control my emotions so as not to hurt the patient”. Responses are provided using a scale ranging from 1 (poor) to 6 (excellent). The scale scores were represented as the average of all items on the scale. Higher scores reflect higher levels of ethical behavior in nursing. Cronbach’s α in Ode’s study was 0.80, and in the current study it was 0.90.

#### 2.2.7. Self-Efficacy of Discharge Planning for End-Stage Cancer Patients

Self-efficacy regarding discharge planning for end-stage cancer patients was measured using the VAS. The participants were asked to indicate their degree of confidence in discharge planning for patients with end-stage cancer. Higher scores indicated greater confidence to practice discharge planning in ward nurses (0 mm = “no confidence” to 100 mm = “extreme confidence”).

#### 2.2.8. Practice of Discharge Planning for End-Stage Cancer Patients

Discharge planning practice for end-stage cancer patients was assessed using the self-reported Discharge Planning of Ward Nurses (DPWN) scale developed by Sakai et al. [14]. The scale was developed to measure general discharge planning practice in nurses who do not specialize in working with patients with end-stage cancer. We instructed participants to evaluate cases involving patients with end-stage cancer with the original authors’ permission. Therefore, we conducted an exploratory factor analysis to examine whether the structure of the scale in the current study was the same as that of the original scale. The scale includes 24 items, with responses provided using a scale ranging from 1 (poor) to 6 (excellent). Items are divided between four subscales (providing discharge guidance in cooperation with a community support team and multidisciplinary team; collecting information from the client/family; assisting with use of social resources; and supporting the decision-making process). Total scores are calculated as the sum of the item scores (range: 24–144). Higher scores reflect nurses’ higher self-evaluation of discharge planning practice for patients with end-stage cancer. Cronbach’s α in the current study was 0.96.

### 2.3. Data Analysis

The data that had missing values on scales were excluded. For the analysis, death discussion with patients and interprofessional support were dichotomized using ratings of 1 and 2 combined into a score of 1 (No), and 3 and 4 combined into a score of 0 (Yes); lack of time was dichotomized using ratings of 1 and 2 combined into a score of 1 (Yes), and 3 and 4 combined into a score of 0 (No). We checked the distribution of the data for skewness and kurtosis before conducting the analyses. The kurtosis of do-no-harm, do-good was over 2.0; therefore, the nonparametric method was used to conduct univariate analysis. If the skewness is over 2.0 and the kurtosis is over 7.0 in absolute values, they will have a substantial influence on the estimation result in structural equation modeling [38]. All scale values fell within these criteria and were accordingly included in the path analysis.

We conducted descriptive statistics for each value. After that, we performed univariate analysis using Spearman’s rank correlation to assess the relationship between the study variables, providing clues on how the variables may affect each other in the model. An exploratory path analysis was conducted to test the fit of the hypothetical model. The data-model fit was examined using several goodness of fit indices: the *p* value of the chi-square test > 0.05, CFI > 0.95 [39], GFI > 0.90, AGFI > 0.90, and RMSEA < 0.06 [39]. Mardia’s coefficient was 9.27, which indicated non-multivariate normality. Therefore, the path coefficient estimation and effect analysis were conducted using the generalized least squares method, which does not assume non-multivariate normality. The bootstrapping method was used to verify the significance of the direct, indirect, and total effects. The significance level was set at *p* < 0.05 in univariate analysis. Data were analyzed using the IBM SPSS statistics for windows software version 26.0 (IBM Corp., Armonk, NY, USA) and AMOS 26.0 (IBM Corp., Armonk, NY, USA).

### 2.4. Ethical Consideration

The study was conducted after receiving approval from the Ethics Committee of the Faculty of Health Sciences of Hokkaido University (18–40). The participants received a written explanation of the study aims, methods, protection of their information and privacy, voluntary participation, and publication of the results. Participants’ submission of the questionnaire was considered as their consent.

## 3. Results

### 3.1. Participants’ Characteristics

In total, 348 general ward nurses returned the questionnaire (73.3% response rate) and 288 valid responses that had no missing values in the scale items were analyzed (82.8% valid response rate). The nurses’ descriptive characteristics are shown in Table 1. The participants’ mean age was 33.42 years (SD = 8.97). Most nurses were female (273; 94.8%), had a diploma (216; 75.0%), and had no experience of homecare or DPNs (265; 92.0%).

### 3.2. Attitude, Nursing Experience, Knowledge, Perceived Skills, Perceived Barriers, Nursing Ethics, Self-Efficacy, and Practice of Discharge Planning for End-Stage Cancer Patients

Table 2 shows results of description statistics of variables. Regarding attitude, the mean score for the importance of discharge planning for end-stage cancer patients was 82.85 (SD = 17.79) and the mean score for the degree of leaving it to DPNs was 60.56 (SD = 18.86).

The mean number of years of nursing experience was 10.86 (SD = 8.46). Regarding knowledge, 43.4% of the participants had experience of attending homecare seminars.

Regarding perceived skills, the score for assertiveness was 3.66 (SD = 0.89), other acceptance was 4.57 (SD = 0.81), and regulation of interpersonal relationships was 4.27 (SD = 0.79).

Regarding perceived barriers, 38.2% of the participants were not able to discuss death with end-stage cancer patients, 83.0% felt a lack of time to prepare for discharge planning, and 20.8% had less interprofessional support.

Regarding nursing ethics, the score for do-no-harm, do-good was 4.37 (SD = 0.57). The mean score for self-efficacy of discharge planning was 29.24 (SD = 19.29). The mean score of the practice of discharge planning for end-stage cancer patients was 87.35 (SD = 14.04).

### 3.3. Univariate Analysis

Table 3 shows the correlations among variables. Self-efficacy had correlations with the degree of leaving it to DPNs (rs = −0.168, *p* < 0.01), years of experience (rs = 0.377, *p* < 0.001), experience of attending home care seminars (rs = 0.346, *p* < 0.001), assertiveness (rs = 0.312, *p* < 0.001), regulation of interpersonal relationships (rs = 0.156, *p* < 0.01), and death discussion (rs = −0.365, *p* < 0.001). However, self-efficacy had no significant correlations with importance, other acceptance, lack of time, interprofessional support, and do-no-harm, do-good.

Practices of discharge planning for end-stage cancer patients had correlations with importance (rs = 0.119, *p* < 0.005), degree of leaving it to DPNs (rs = −0.183, *p* < 0.01), years of nursing experience (rs = 0.339, *p* < 0.001), experience of attending home care seminars (rs = 0.358, *p* < 0.001), assertiveness (rs = 0.373, *p* < 0.001), other acceptance (rs = 0.203, *p* < 0.01), regulation of interpersonal relationships (rs = 0.276, *p* < 0.001), death discussion (−0.359, *p* < 0.001), lack of time (rs = −0.128, *p* < 0.05), interprofessional support (rs = −0.132, *p* < 0.05), do-no-harm, do-good (rs = 0.305, *p* < 0.001), and self-efficacy (rs = 0.574, *p* < 0.001).

### 3.4. Path Analysis

**Figure 2:**
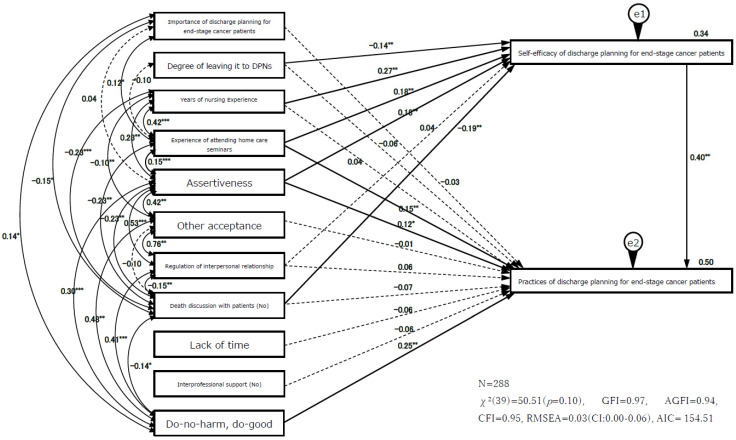
Path analysis of hypothesized model. Note: Dotted line is nonsignificant path, *: *p* < 0.05, **: *p* < 0.01, ***: *p* < 0.001.

Based on the hypothesized model and significant relationships found from the univariate analysis, all the variables were used to construct the model (Figure 2). This model’s fit indices were as follows: chi-square χ^2^ (39) = 50.51 (*p* = 0.10), GFI = 0.97, AGFI = 0.94, CFI = 0.95, and RMSEA = 0.03 (CI: 0.00–0.06).

Table 4 shows the standardized direct, indirect, and total effects of the exogenous and endogenous variables. Self-efficacy was influenced by years of nursing experience (β = 0.27, *p* = 0.003); death discussion with patients (β = −0.19, *p* = 0.003); assertiveness (β = 0.18, *p* = 0.002); experience of attending home care seminars (β = 0.18, *p* = 0.010); and degree of leaving it to DPNs (β = −0.14, *p* = 0.007). The variance of self-efficacy in the model accounted for 34%.

As hypothesized, self-efficacy was closely related to practices of discharge planning for end-stage cancer patients (β = 0.40, *p* = 0.002) and mediated the relationship between distal factors and practices of discharge planning for end-stage cancer patients. Self-efficacy mediated the relationships of years of nursing experience (β = 0.11, *p* = 0.002), death discussion with patients (β = −0.08, *p* = 0.002), assertiveness (β = 0.07, *p* = 0.002), experience of attending home care seminars (β = 0.07, *p* = 0.006), and degree of leaving it to DPNs (β = −0.06, *p* = 0.005) with practices of discharge planning for end-stage cancer patients. Experience of attending home care seminars (β = 0.15, *p* = 0.003) and assertiveness (β = 0.12, *p* = 0.044) also had a significant direct effect on practices of discharge planning for end-stage cancer patients. Do-no-harm, do-good had a direct influence on practices of discharge planning for end-stage cancer patients (β = 0.25, *p* = 0.001), without self-efficacy as the mediator.

Regarding the total effect on practices of discharge planning for end-stage cancer patients, self-efficacy (β = 0.40, *p* = 0.003); do-no-harm, do-good (β = 0.25, *p* = 0.001); experience of attending home care seminars (β = 0.22, *p* = 0.002); assertiveness (β = 0.20, *p* = 0.001); years of nursing experience (β = 0.15, *p* = 0.013); death discussion with patients (β = −0.14, *p* = 0.002) and degree of leaving to DPNs (β = −0.12, *p* = 0.010) had significant effects. The variance of the practice of discharge planning for end-stage cancer patients in this model accounted for 50%.

## 4. Discussion

To our knowledge, this study is the first to show self-efficacy and nurses’ ethical behavior directly and positively influencing their practice of discharge planning for end-stage cancer patients. This study examined general ward nurses’ practices of discharge planning for end-stage cancer patients, using the self-efficacy theory as a constructive framework. Our constructed model in this study was confirmed by path analysis to have a superior fit. This was clarified through the relationships among the factors influencing nurses’ practices of discharge planning for end-stage cancer patients. The results partially supported our overall hypotheses. Self-efficacy, do-no-harm, do-good, experience of attending home care seminars, and assertiveness directly and positively influenced the practice of discharge planning for end-stage cancer patients in that order. Years of nursing experience, assertiveness, and attending home care seminars positively influenced, while lack of death discussion and the degree of leaving it to DNPs negatively influenced, the practice of discharge planning with self-efficacy as the mediator.

### 4.1. Self-Efficacy

As we hypothesized, self-efficacy was the central factor in this study. It most directly and positively influenced nurses’ practice of discharge planning for end-stage cancer patients. This result reflected Bandura’s theory that perceived self-efficacy influences a person’s behavior [20] and was similar to the findings of previous studies examining the relationship between self-efficacy and nurses’ practice [22,24]. Self-efficacy also mediated the relationships of years of nursing experience, death discussion, assertiveness, experience of attending home care seminars, and degree of leaving it to DPNs on nurses’ practice of discharge planning for end-stage cancer patients. This mediating effect of self-efficacy in the relationships between other factors and nursing practice resembles the results of a prior study, which found that self-efficacy played a mediating role in nurses’ weight management practice [25]. These results show that enhancing nurses’ self-efficacy regarding discharge planning for cancer patients increases their discharge planning practice and that improving nurses’ death discussion, communication skills, and attitude of not leaving it to DNPs would contribute to it.

The participants’ mean self-efficacy score in this study was 29.24 (SD = 19.29). Since this is the first study, to the best of our knowledge, that measures nurses’ self-efficacy of discharge planning for end-stage cancer patients, our data are not directly comparable. However, we can say that the VAS score for this study was low. Considering that years of nursing experience and assertiveness positively influenced self-efficacy, confidence as a nurse might have an influence on the evaluation of self-efficacy of the practice of discharge planning for end-stage cancer patients. It has been reported that Japanese people are hesitant to evaluate their performance as better [40]. Similar to this, Japanese nurses might tend to evaluate their performance as low and have lower confidence as nurses. This tendency would possibly affect the low score of self-efficacy of discharge planning for end-stage cancer patients. Though further study is needed to confirm this, our results suggest that efforts to enhance nurses’ self-efficacy are necessary by improving death discussions, communication skills, and attitude to discharge planning through education and encouragement to attend home care seminars.

### 4.2. Nurses’ Ethiccal Behavior

As mentioned in our hypothesis, do-no-harm, do-good had a direct influence on the practice for discharge planning for end-stage cancer patients. This result indicates that nurses who recognize the importance of beneficence and nonmaleficence have better practice than those who do not. This is a novel finding in our study. Nursing ethics comprises nurses’ codes of conduct that they follow. In end-of-life care, autonomy is emphasized as an essential component [41]. However, this study shows that beneficence and nonmaleficence are also important concepts in end-of-life care discharge planning, which should involve respect for patients’ autonomy. Furthermore, the fact that do-no-harm, do-good directly influenced nurses’ practice for discharge planning for end-stage cancer patients, regardless of self-efficacy, was notable. In this study, participants’ average level of self-efficacy regarding discharge planning was low. It would thus be necessary for nurses to behave based on ethical beliefs of beneficence and nonmaleficence and to practice discharge planning by overcoming the barriers and low confidence.

These findings indicate the importance of enhancing nurses’ perception and behaviors of complying with beneficence and nonmaleficence to improve their discharge planning practice. Nishimura [42] reported that nurses who had ethics education, training, or conference experience displayed more ethical behavior. These results indicate the possibility that education improves nurses’ ethical behavior. Suzuki et al. [43] reported the effects of discharge planning educational programs designed based on the KAP (knowledge, attitude, and practice) model. In the discharge planning of end-stage cancer patients, ethical aspects including beneficence and nonmaleficence should also be included in the educational program.

### 4.3. Nursing Experience and Knowledge

Years of nursing experience directly influenced nurses’ self-efficacy and indirectly influenced their practice of discharge planning. This shows that nurses with longer experience have higher self-efficacy and practice of discharge planning. Bandura [20] stated that performance accomplishment is especially influential in the four sources of efficacy information, and self-efficacy is developed by repeated success. The nurses that have a long experience might have more successful experiences with discharge planning for end-stage cancer patients than those who have a short experience.

Experience of attending home care seminars influenced self-efficacy, which, in turn, influenced the practice of discharge planning for end-stage cancer patients in this study. Previous studies [29,44,45] have reported that education programs increase individual self-efficacy, as with our results. Japanese home care laws and systems are difficult to understand, with the system being reviewed and changed every few years. Therefore, nurses would need to attend home care seminars after their graduation to practice discharge planning effectively and with confidence.

### 4.4. Barriers

Lack of death discussion with patients influenced self-efficacy directly and the practice of discharge planning indirectly. This result indicates that nurses who cannot discuss death with end-stage cancer patients have lower self-efficacy and less practice of discharge planning. Nurses might need to discuss death with patients to understand how and where they would like to spend their limited time. The understanding of patients’ desires clarifies the direction of discharge planning for the nurses. Without this conviction of care direction, nurses’ self-efficacy and practice of discharge planning would not rise. Previous studies show that nurses have difficulties in discussing death with patients. The reasons for this include nurses’ death anxiety [46], difficulty in handling patients’ questions [47,48], lack of communication skills [49], and cultural taboo [48,50,51]. In Japan, there is also a taboo surrounding the mention of death, like in other Asian countries. However, as the importance of self-determination in the end-stage has been enhanced by the Ministry of Health, Labor and Welfare, in Japan [52,53], patients’ and nurses’ recognition of death discussion is changing. To perform discharge planning with confidence, it would be necessary to decrease nurses’ aversion to discussing death with end-stage patients.

### 4.5. Perceived Skills

Assertiveness positively and directly influenced self-efficacy and influenced the practice of discharge planning both directly and indirectly. In other words, nurses with higher communication skills of assertiveness would have higher self-efficacy and greater practice of discharge planning. In the discharge planning process, nurses must coordinate and collaborate with patients, their families, and healthcare professionals in both the hospital and home care setting. Previous studies reported that these collaborations are challenging issues for nurses, with patients, their families, and interprofessional relationships sometimes involving different opinions on discharge planning [54,55]. In such situations, nurses should play an advocating role to support patients’ decision making in the discharge planning process. Nurses have to state their opinions to patients, their families, and other professionals to realize discharge planning in line with the patients’ wishes. This demands that nurses have effective communication skills of assertiveness [56] to have more confidence in discharge planning.

### 4.6. Attitude

Nurses’ attitude was measured as the importance of discharge planning and degree of leaving it to DPNs. There were no significant relationships between the importance of discharge planning and self-efficacy and the practice of discharge planning in the model. In this study, the mean score of the importance of discharge planning was over 80, which was high, and these results were similar to those of a previous study [12]. Most participants recognized discharge planning as an important aspect and, therefore, gave high scores, which might be the reason for a nonsignificant difference. Moreover, there may be a situation in which nurses have to practice discharge planning regardless of the recognition of importance they have.

On the other hand, the degree of leaving it to DPNs negatively and directly influenced self-efficacy, and influenced the practice of discharge planning indirectly. This indicates that nurses who have a passive attitude have lower self-efficacy and practice of discharge planning for end-stage cancer patients. The reason why nurses want to leave it to DPNs might be a lack of recognition that discharge planning is their role. Additionally, there might be a lack of understanding of role sharing between general ward nurses and DPNs as per a previous study [12]. It would be necessary to promote nurses’ positive attitude toward the practice of discharge planning for end-stage cancer patients and nurses’ understanding of role sharing between general ward nurses and DPNs.

### 4.7. Strengths and Limitations

This study is the first to show the relationship between self-efficacy and ethical behavior with discharge planning. It is also unique in that it assesses the hypotheses using the path model of interrelationships between six factors (attitude, knowledge and nursing experience, perceived skills, perceived barriers, nurses’ ethical behavior, and self-efficacy) and practices of discharge planning for end-stage cancer patients. The model was well fitted and the explanation rate was good.

However, there are some limitations in this study. First, we used the VAS scale to measure self-efficacy because proper scales did not exist. It is desirable to develop an instrument that can measure the self-efficacy of discharge planning and that has good reliability and construct validity. Second, the participants in this study consisted of only nurses who were working at hospitals which were designated as facilities to promote home-based cancer and palliative care in one prefecture in Japan. Thus, the generalization of the findings may be limited. Third, the data were collected through a self-administered questionnaire. This can cause bias, such as social desirability. Finally, this study could not establish causal relationships between the variables because a cross-sectional study design was used.

## 5. Conclusions

To our knowledge, this study is the first to show self-efficacy and nurses’ ethical behavior directly and positively influencing their practice of discharge planning for end-stage cancer patients. Self-efficacy was the most influential factor on nurses’ practice of discharge planning, mediating the relationship between other factors (degree of leaving it to DNPs, years of nursing experience, experience of attending home care seminars, assertiveness, and death discussion) and the practice of discharge planning. Furthermore, do-no-harm, do-good, experience of attending home care seminars, and assertiveness directly influenced the practice of discharge planning. Only do-no-harm, do-good influenced nurses’ practice without the mediating effect of self-efficacy. This study’s results suggest the need to enhance self-efficacy and nursing ethical behavior among general ward nurses. In order to do that, training and education about home care, assertive communication skills, death discussion, and ethics would be needed for this population.

## Figures and Tables

**Table 1:** Participants’ characteristics (N = 288).

Characteristics	*n* (%)
Gender	
Male	15 (5.2)
Female	273 (94.8)
Educational background	
Bachelor’s degree	63 (21.9)
Associate degree	8 (2.8)
Diploma	216 (75.0)
Missing	1 (0.3)
Ward setting	
Medical ward or Med/Surge ward	242 (84.0)
Surgical ward	46 (16.0)
Experience of homecare or DPNs	
No	265 (92.0)
Yes	23 (8.0)
	M ± SD
Age	33.42 ± 8.97

**Table 2:** Results of description statistics (N = 288).

Variables		Items	Mean ± SD/*n* (%)	Min	Max	Possible Range	Cronbach’s α
Attitude							
Importance of discharge planning for end-stage cancer patients (VAS scale)			82.85 ± 17.79	14.00	100.00	0–100	
Degree of leaving to DPNs (VAS scale)			60.56 ± 18.86	9.00	100.00	0–100	
Nursing experience and knowledge							
Years of nursing experience			10.86 ± 8.46	0	37	-	
Experience of attending home care seminars	No = 0		163 (56.6)				
	Yes = 1		125 (43.4)				
Perceived skills							
Assertiveness		4	3.66 ± 0.89	1.00	7.00	1–7	0.89
Other acceptance		4	4.57 ± 0.81	1.00	7.00	1–7	0.94
Regulation of interpersonal relationship		4	4.27 ± 0.79	1.00	7.00	1–7	0.87
Perceived barriers							
Death discussion with patients	No = 1		110 (38.2)				
	Yes = 0		178 (61.8)				
Lack of time	No = 0		49 (17.0)				
	Yes = 1		239 (83.0)				
Interprofessional support	No = 1		60 (20.8)				
	Yes = 0		228 (79.2)				
Nurses’ ethical behavior							
Do-no-harm, do-good		9	4.37 ± 0.57	1.00	5.89	1–6	0.90
Self-efficacy of discharge planning for end-stage cancer patients (VAS scale)			29.24 ± 19.29	0.00	100.00	0–100	
Practice of discharge planning for end-stage cancer patients		24	87.35 ± 14.04	49.00	130.00	24–144	0.96

**Table 3:** Correlations among variables (N = 288).

Variables	1	2	3	4	5	6	7	8	9	10	11	12	13
1 Importance of discharge planning for end-stage cancer patients	1.000												
2 Degree of leaving it to DPNs	0.012	1.000											
3 Years of nursing experience	0.027	−0.058	1.000										
4 Experience of attending home care seminars	0.133 *	−0.130 *	0.491 ***	1.000									
5 Assertiveness	0.126 *	−0.022	0.251 ***	0.152 *	1.000								
6 Other acceptance	0.021	0.002	−0.116 *	0.037	0.353 ***	1.000							
7 Regulation of interpersonal relationships	0.079	−0.016	−0.010	0.034	0.491 ***	0.727 ***	1.000						
8 Death discussion with patients (No)	−0.169 **	0.078	−0.225 ***	−0.241 ***	−0.263 ***	−0.150 *	−0.204 **	1.000					
9 Lack of time	0.094	−0.104	0.089	0.005	0.005	−0.007	−0.065	0.033	1.000				
10 Interprofessional support (No)	−0.060	−0.056	0.053	−0.052	−0.054	−0.075	−0.081	0.054	−0.073	1.000			
11 Do-no-harm, do-good	0.122 *	−0.051	−0.024	0.005	0.250 ***	0.455 ***	0.382 ***	−0.179 **	−0.065	−0.114	1.000		
12 Self-efficacy of discharge planning for end-stage cancer patients	0.043	−0.168 **	0.377 ***	0.346 ***	0.312 ***	0.015	0.156 **	−0.365 ***	−0.094	−0.047	0.013	1.000	
13 Practices of discharge planning for end-stage cancer patients	0.119 *	−0.183 **	0.339 ***	0.358 ***	0.373 ***	0.203 **	0.276 ***	−0.359 ***	−0.128 *	−0.132 *	0.305 ***	0.574 ***	1.000

Spearman’s rank correlation coefficient, *: *p* < 0.05, **: *p* < 0.01, ***: *p* < 0.001.

**Table 4:** The model standardized direct, indirect, and total effect (N = 288).

Endogenous Variables	Direct Effect (*p*)	Bias-Corrected 95% CI	Indirect Effect (*p*)	Bias-Corrected 95% CI	Total Effect (*p*)	Bias-Corrected 95% CI
Exogenous Variables						
Self-efficacy of discharge planning for end-stage cancer patients						
Degree of leaving to DPNs	−0.14 (0.007)	(−0.293, −0.039)	-	-	-	-
Years of nursing experience	0.27 (0.003)	(0.140, 0.398)	-	-	-	-
Experience of attending home care seminars	0.18 (0.010)	(0.057, 0.286)	-	-	-	-
Assertiveness	0.18 (0.002)	(0.065, 0.310)	-	-	-	-
Regulation of interpersonal relationship	0.04 (0.532)	(−0.088, 0.163)	-	-	-	-
Death discussion with patients (No)	−0.19 (0.003)	(−0.290, −0.081)	-	-	-	-
Practice of discharge planning for end-stage cancer patients						
Importance of discharge planning for end-stage cancer patients	−0.03 (0.525)	(−0.127, 0.060)	0.00	-	−0.03 (0.525)	(−0.127, 0.060)
Degree of leaving to DPNs	−0.06 (0.118)	(−0.155, 0.014)	−0.06 (0.005)	(−0.124, −0.018)	−0.12 (0.010)	(−0.234, −0.029)
Years of nursing experience	0.04 (0.407)	(−0.058, 0.139)	0.11 (0.002)	(0.053, 0.175)	0.15 (0.013)	(0.040, 0.250)
Experience of attending home care seminars	0.15 (0.003)	(0.057, 0.255)	0.07 (0.006)	(0.027, 0.124)	0.22 (0.002)	(0.117, 0.324)
Assertiveness	0.12 (0.044)	(0.004, 0.242)	0.07 (0.002)	(0.025, 0.127)	0.20 (0.001)	(0.078, 0.332)
Other acceptance	−0.01 (0.956)	(−0.155, 0.145)	0.00	-	−0.01 (0.956)	(−0.155, 0.145)
Regulation of interpersonal relationship	0.06 (0.408)	(−0.089, 0.215)	0.02 (0.529)	(−0.034, 0.070)	0.08 (0.340)	(−0.081, 0.226)
Death discussion with patients (No)	−0.07 (0.171)	(−0.160, 0.024)	−0.08 (0.002)	(−0.129, −0.034)	−0.14 (0.002)	(−0.247, −0.048)
Lack of time	−0.06 (0.143)	(−0.144, 0.022)	0.00	-	−0.06 (0.143)	(−0.144, 0.022)
Interprofessional support (No)	−0.06 (0.164)	(−0.138, 0.026)	0.00	-	−0.06 (0.164)	(−0.138, 0.026)
Do-no-harm, do-good	0.25 (0.001)	(0.171, 0.360)	0.00	-	0.25 (0.001)	(0.171, 0.360)
Self-efficacy of discharge planning for end-stage cancer patients	0.40 (0.002)	(0.287, 0.499)	0.00	-	0.40 (0.002)	(0.287, 0.499)

## Data Availability

The data presented in this study are available upon request from the corresponding author. The data are not publicly available due to privacy.
